# A Novel Insulinotropic Peptide from the Skin Secretions of *Amolops loloensis* Frog

**DOI:** 10.1007/s13659-014-0037-z

**Published:** 2014-10-08

**Authors:** Guo-Xiang Mo, Xue-Wei Bai, Zong-Jie Li, Xiu-Wen Yan, Xiao-Qing He, Ming-Qiang Rong

**Affiliations:** 1 School of Biological Sciences, Nanjing Agriculture University, Nanjing, 210095 Jiangshu China; 2 Kunming Institue of Zoology, Chinese Academy of Sciences, Kunming, 650223 Yunnan China

**Keywords:** Insulinotropic peptide, *Amolops loloensis*, Skin secretion, Insulin-releasing, Frog

## Abstract

Various kinds of biologically active peptides have previously been isolated from the skin secretions of *Amolops loloensis* frog, such as antimicrobial peptides, bradykinin-like peptides and algesic peptides. A novel insulinotropic peptide named amolopin was identified in *A. loloensis* frog’s skin secretion. Its primary structure sequence was determined by Edman degradation as: FLPIVGKSLSGLSGKL-NH2. BLAST search indicates that the amino acid sequence of amolopin is quite different from other known insulin secretagogues, including mastoparan, exendins and α-latrotoxin, nor does it like incretins (e.g. glucagons like peptide-1 and glucose-dependent insulinotropic ploypeptide) either. However, amolopin shows certain structural similarity with amphibian antimicrobial temporins and vespid chemotactic peptides isolated from *Vespa magnific*a. Amolopin can stimulate insulin release in INS-1 cells in a dose-dependent manner. Primary investigation on its action mechanisms reveals that amolopin does not increase the influx of Ca^2+^. In conclusion, a novel 16-amino acid peptide with insulin-releasing activity is initially discovered from the skin secretion of *A. loloensis* frog. Further work is necessary to evaluate its potential as novel anti-diabetic candidate.

## Introduction

Amphibian skin granular gland secretions are consistently recognized as rich sources of multiple biologically active compounds [1], such as antimicrobial peptides (AMPs) [2], bradykinins, biogenic amines, complex alkaloids, etc. These compounds are of great importance for the amphibians to regulate their physiological balance, to resist infection by microorganism, and to escape from being preyed upon by natural predators.

Insulinotropic peptides are various kinds of protein molecules that can stimulate insulin secretion by the pancreatic islets β-cells through different pathways. Over the past decades, several families of insulinotropic peptides have been discovered in lizard venoms [3], snake venoms [4], wasp venoms [5], the black widow spider toxins [6] and so forth. Exentides (exentide-1, -2, -3 and -4) isolated from lizard venoms belong to the glucagon superfamily peptides, most of which are structurally homologous and can increase insulin release, including glucagon-like peptide 1, glucose-dependent insulinotropic polypeptide, pituitary adenylate cyclase-activating polypeptide, vasoactive intestinal peptide and other members. The glucagon superfamily peptides are usually single-chain peptides consisting of 27–39 amino acids. However, insulin-releasing peptides purified from snake venoms and spider venoms (e.g. PLA_2_ and α-LTX) are commonly much larger polymers with molecular weights around 12–130 kDa, which are assembled of several subunits [7, 8]. Mastoparans and temporins are the smallest insulinotropic peptides obtained respectively from wasp venoms and amphibian skin secretions. Native mastoparan is a tetradecapeptide stimulating insulin secretion at a late stage in the secretary pathway [9, 10]. While temporins, the smallest antibacterial peptides from the skin of *Rana temporaria*, bear insulin-releasing activity with a length of merely 10–13 amino acids [11, 12].

As the work of isolating novel insulinotropic peptides and characterizing the insulin-releasing activity of known bioactive peptides are focused on, several new insulinotropic peptides have been isolated from skin secretions of amphibian [13–16]. Simultaneously, due to our previous study, the skin secretions of frog *Amolops loloensis* comprised multiple kinds of biologically active polypeptides, including bradykinin-like peptide [17], antibacterial peptides [18] and algesic peptides [19]. It is very interesting to identify new insulinotropic peptides in *A. loloensis*’s skin secretion and assess their acting mechanism. In this paper, we elucidated the isolation and structural characterization of novel insulinotropic peptides in the skin secretion of rufous-spotted torrent frog, *A. loloensis*.

## Materials and Methods

### Collection of Frog Skin Secretions

Adult specimens of *A. loloensis* of both sexes (*n* = 30; weight range 30–40 g) were collected in Yunnan Province of China. Skin secretions were collected as the following: frogs were put into a cylinder container. A piece of absorbent cotton immersed with anhydrous ether was put on the top of the container. The container was covered with a lid and permeated with volatilized anhydrous ether. Being stimulated by anhydrous ether for 1–2 min, frog skin surface was seen to exude copious secretions. Skin secretions were collected by washing the dorsal region of each frog with 0.1 M NaCl solution (containing 0.01 M EDTA). The collected solutions (500 mL of total volume) were quickly centrifuged and the supernatants were lyophilized.

### Peptide Purification

Lyophilized skin secretion sample of *A. loloensis* (1.5 g, total OD_280 nm_ of 400) was dissolved in 10 mL 0.1 M phosphate buffer, pH 6.0, containing 5 mM EDTA. The sample was applied to a Sephadex G-50 (Superfine, Amersham Biosciences, 2.6 × 100 cm) gel filtration column equilibrated with 0.1 M phosphate buffer, pH 6.0. Elution was performed with the same buffer, collecting fractions of 3.0 mL. The absorbance of the elute was monitored at 280 nm. The insulin-releasing activities of fractions were determined as indicated below. The protein peaks revealed insulin-releasing activity were pooled, lyophilized, and re-suspended in 2 mL 0.1 M phosphate buffer solution, pH 6.0, and purified further by C_18_ reverse phase high performance liquid chromatography (RP-HPLC, Hypersil BDS C_18_, 30 0.46 cm) column.

### Structural Analysis

Complete peptide sequencing was undertaken by Edman degradation on an Applied Biosystem pulsed liquid-phase sequencer, model 491. Fast atom bombardment (FAB) mass spectrometry was carried out on an Autospec-3000 spectrometer, equipped with a high field magnet, using glycerol: 3-nitrobenzylalcohol: dimethylsulphoxide (1:1:1,v:v:v) as mixed matrix. The ion gun was operated at 25 kV with a current of 1 μA using Cs^+^ as the bombarding gas.

### Insulin Secretion Assay

The rat insulinoma INS-1 cells were cultured in RPMI-1640 medium containing 1 mM d-glucose and supplemented with 10 % fetal calf serum, 100 U/mL penicillin, 100 mg/mL streptomycin, 10 M HEPES, 2 M l-glutamine, 1 M sodium pyruvate, and 50 μM β-mercaptoethanol [20]. Culture medium was replaced every 2–3 days. After cells grew to near confluence, they were treated with 0.25 % trypsin and 0.02 % EDTA for 5 min and replated on 25-cm^2^ flasks.

The insulin secretion assay method was modified as previously reported [13, 21]. INS-1 cells were harvested with trypsin/EDTA, seeded into 24-well plates at a density of 3–5 × 10^5^ cells/mL, and allowed to attach overnight. Before insulin-releasing assay, cells were preincubated in 1 mL Krebs–Ringer bicarbonate (KRB) buffer without glucose (115 mM NaCl, 4.7 mM KCl, 1.28 mM CaCl_2_, 1.2 mM KH_2_PO_4_, 1.2 mM MgSO_4_, 10 mM NaHCO_3_ and 1 g/L BSA, pH 7.4) at 37 °C for 45 min. Cells were incubated in the absence or presence of peptide venoms for 1 h, using the same buffer supplemented with 2.8 mM glucose. This glucose concentration enabled to detect the basal condition of insulin secretion. The insulin supernatants were collected, stored at −20 °C, and subsequently determined by radioimmunoassay.

### Measurement of [Ca^2+^]_i_

To confirm whether the stimulatory mechanism of insulin secretion was via the activation of voltage-dependant channel Ca^2+^ (VDCC, the cytosolic concentration of Ca^2+^ was measured with fluorescent Ca^2+^ probe Fluo-3/AM **(**Dojindo Laboratories, Japan**)** according to formerly described [22–24]. INS-1 cells were plated onto glass bottom dishes (MatTek Corporation) and cultured overnight, and then 5 µM/DMSO Fluo-3/AM plus Pluronic F127 was added. The cells were loaded for 30 min, washed three times with HEPES buffer saline (10 mM HEPES, 1 mM Na_2_HPO_4_, 137 mM NaCl, 5 mM KCl, 1 mM CaCl_2_, 0.5 mM MgCl_2_, 5 mM glucose, 0.1 % BSA, pH 7.4), and scanned with a confocal laser fluorescent microscope (40× objective lens). The fluorescence was excited at 480 nm at 10-s intervals and detected at 515 nm, whose intensity were imaged after incubated with peptide venoms to indicate the [Ca^2+^]_i_ of individual cells.

### Statistical Analysis of the Data

The statistical analysis of the data was carried out by SPSS statistical software. The results were expressed as the mean ± SEM of five experiments. While comparing the change, the data was analyzed by one-way ANOVA, followed by the LSD to detect significant difference between different groups. The level of significance was set at *P* < 0.05.

## Results

### Purification of Insulinotropic Peptide

The supernatant of *A. loloensis* skin secretions was divided into five peaks by Sephadex G-50 as report in our previous work [25]. A insulin-releasing activity peptide was firstly found and then an RP-HPLC column was used in order to get the purified insulin–releasing activity was peptide.

### Structural Analysis

**Fig. 1:**
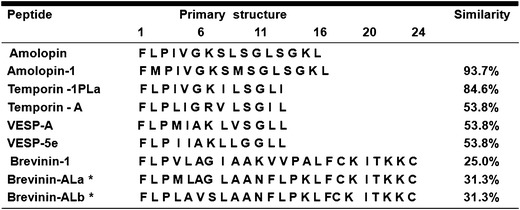
The amino acid sequence comparison of amolopin with other bioactive peptides. Peptide amolopin is from this study, amolopin-1 from *Amolops loloensis* [25], temporin-1PLa from *Rana palustris* [31], temporin-A from *Rana temporaria* [30], VESP-A and VESP-5e from *Vespa magnifica* [26], brevinin-1 from *Rana brevipoda porsa* [29] and brevinin-ALa, -ALb also from *Amolops loloensis* [18]

The purified insulinotropic peptide designated as amolopin was subjected to amino acid sequence analysis by automated Edman degradation. The amino acid sequence of amolopin was determined as: FLPIVGKSLSGLSGKL-NH2. This 16-amino acid peptide includes a C-terminally amidated residue. Its molecular weight analyzed by FAB mass spectrometry was 1615.5 Da, which was nearly identical to the theoretical molecular weight, 1615.98 Da. BLAST search showed that amolopin was quite different from other known insulinotropic peptides, e.g. mastoparans, the glucagon superfamily peptides, PLA_2_ from snake venoms, and α-LTX from black widow spider venoms (Fig. 1). This revealed a new insulinotropic peptide. Analysis by the ExPASy MW/pI tool (http://www.expasy.org/tools/pi_tool.html) indicated its predicted pI was 10. As shown in Fig. 1, amolopin shows certain homology with brevinins and temporins AMPs from amphibian skin as well as vespid chemotactic peptides from vespid venoms [26].

### Insulin Secretion

**Fig. 2:**
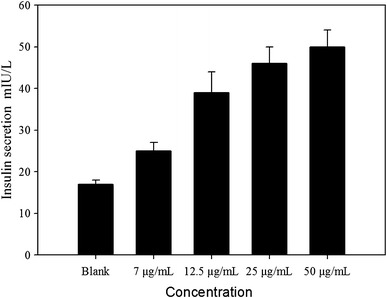
The iusulinotropic effect of amolopin. Amolopin revealed obvious insulin-releasing activity on rat insulinoma INS-1 cells. The stimulation was quantified after 1 h incubation with peptide sample, compared with KRB buffer alone. The values are the means of ±SEM for five independent experiments

As shown in Fig. 2, amolopin exhibited markedly insulin-releasing activity on rat insulinoma INS-1 cells. After incubation for 1 h in 1 mL KRB buffer (115 mM NaCl, 4.7 mM KCl, 1.28 mM CaCl_2_, 1.2 mM KH_2_PO_4_, 1.2 mM MgSO_4_, 10 mM NaHCO_3_ and 1 g/L BSA, pH 7.4) at 37 °C. Amolopin augmented insulin secretion by INS-1 cells at different concentrations compared with 2.8 mM glucose alone. The INS-1 cell line was widely used for insulin secretion researches, which was established from radiation induced tumor growing in medium with 2-ME (β-mercaptoethanol) [20]. The INS-1 cells were used between 18 and 25 passages. The stimulatory effects of amolopin enhanced as the peptide concentrations added, and statistical analysis by SPSS 13.0 revealed that amolopin significantly increased insulin release at the concentrations of 12.5, 25 and 50 μg/mL (Fig. 2).

### [Ca^2+^]_i_ Imaging

**Fig. 3:**
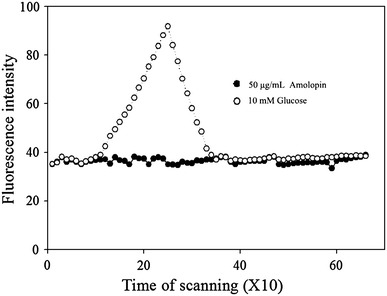
Measurement of [Ca^2+^]_i_ in INS-1 cells. The INS-1 cells were loaded with 5 µM Fluo-3/AM and washed three times with HEPES buffer saline. The cells were incubated in KRB buffer and scanned by a confocal laser microscope for 60 s as blank control. As indicated by the *arrow*, 10 mM glucose and amolopin sample was added and scanned sequentially for another 10 min. [Ca^2+^]_i_ indicated by the fluorescence intensity was measured. No evident increasing change of [Ca^2+^]_i_ and was observed after the addition of amolopin. Data is the means of five independent experiments

The cytosolic Ca^2+^ concentration was measured with fluorescent Ca^2+^ probe Fluo-3/AM to assess whether the insulin-releasing activity was induced by the activation of Ca^2+^ channel and Ca^2+^ influx. After the INS-1 cells were loaded with 10 mM glucose and 5 µM Fluo-3/AM, amolopin samples were added into incubation medium in the glass bottom dishes. The fluorescence indicating [Ca^2+^]_i_ was scanned by a confocal laser fluorescent microscope at 10-s intervals. However, no significant increase in the fluorescence intensity was observed after the amolopin peptide was added. Though Ca^2+^ played a important role in the pathway of insulin exocytosis, this data confirmed amolopin stimulated insulin secretion not via Ca^2+^ influx. Experiments were repeated at least five times with similar results (Fig. 3).

## Discussion

In the present study, a unique16-amino acid insulinotropic peptide termed amolopin was obtained from the skin secretion of *A. loloensis* by gel filtration and RP-HPLC, which was structurally different from other known insulinotropic peptides. *A. loloensis* frog belonged to Ranidae, Amolopinae [27, 28] and inhabited in Southeast Asiain torrent rivulets. Yet, numerous bioactive peptide had already been isolated from the skin secretion of *A. loloensis,* such as AMPs, bradykinin-like peptides and algesic peptides. These components might play a crucial role either in fighting against pathogenic germs, or in repelling dangerous enemies by causing nociceptive responses. However, insulinotropic peptides might also possess approximate defence action through disturbing the physiological functions of their predator’s endocrine organs and inducing hypoglycemia.

FAB mass spectrometry was employed to determine the molecular weight of amolopin and got the result of 1615.5 Da, which was almost identical to the theoretically calculated molecular mass 1615.98 Da by the ExPASy MW/pI tool (http://www.expasy.org/tools/pi_tool.html). Its sequence was assessed through the automated Edman degradation method as: FLPIVGKSLSGLSGKL-NH2. It belongs to the small molecular weight group of insulinotropic peptides together with mastoparans, temporins, and brevinins, distinctive from the large molecular weight group (e.g. PLA_2_ from snake venoms and α-LTX from black widow spider venoms) and the glucagon superfamily peptides. Brevinins are a family of antimicrobial peptides with the length of 24-amino acids initially isolated from the skin of *Rana brevipoda porsa* [29], and are found to have insulin-releasing activity [14, 16]. Temporins are also a large family of antimicrobial peptides with 13-residues firstly identified in the European red frog *Rana temporaria* [11], and other members were subsequently discovered in several North America Rana species, including *R. clamitans, R. luteiventris, R. pipiens, R. grylio, and A. loloensis* and *R. palustris* [18, 30, 31]. As multifunctional molecules, several temporins are proved to display insulin-releasing property [12].

According to Fig. 1, amolopin shares merely a little similarity with partial sequence of brevinins (brevinin-1, 25 %; -ALa, 31.3 %; -ALb, 31.3 %). However, it has certain homology with antimicrobial temporins (temporin-A, 53.8 %; -1PLa, 84.6 %). What’s more interestingly, amolopin is also similar to vespid chemotactic peptides (VESP-A; -5e both 53.8 %), which were detected from vespid venoms [25].

Insulin secretion assay, as illustrated Fig. 2, demonstrated that different concentrations of amolopin could significantly enhance insulin release by INS-1 clonal β-cells (*P* < 0.05), a cell line which was widely used for insulin secretion studies [32]. In order to investigate the stimulatory mechanism of amolopin, [Ca^2+^]_i_ was measured with Fluo-3 and confocal microscope. However, no evident Ca^2+^ influx increase was observed after the addition of amolopin. Therefore, amolopin stimulates insulin secretion probably not via activation of Ca^2+^ channel.

In summary, amolopin is a novel insulinotropic peptide characterized from the skin secretion of *A. loloensis* with distinctive primary structure. It may become a novel and promising anti-diabetic candidate after additional study to verify its efficacy and safety.
